# Supplementary material for: Identification of photoperiod-induced specific miRNAs in the adrenal glands of Sunite sheep (Ovis aries)
Source: Front Vet Sci. 2022 Jul 22;9:888207. doi: 10.3389/fvets.2022.888207 (PMC9354845; doi:10.3389/fvets.2022.888207)
Supplement: Supplementary file 1 [file Data_Sheet_1.ZIP › Supplementary file/miRDeep2 file.zip!/miRDeep2 file/Novel_Structure/LP42A1/Novel_246.pdf]

Provisional ID	:	NC_019472.2_24095
Score total	:	2
Score for star read(s)	:	-1.3
Score for read counts	:	0
Score for mfe	:	1.7
Score for randfold	:	1.6
Score for cons. seed	:	
Total read count	:	211
Mature read count	:	211
Loop read count	:	0
Star read count	:	0

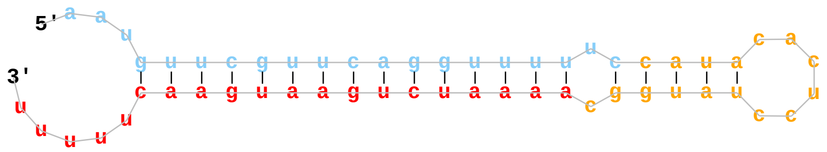

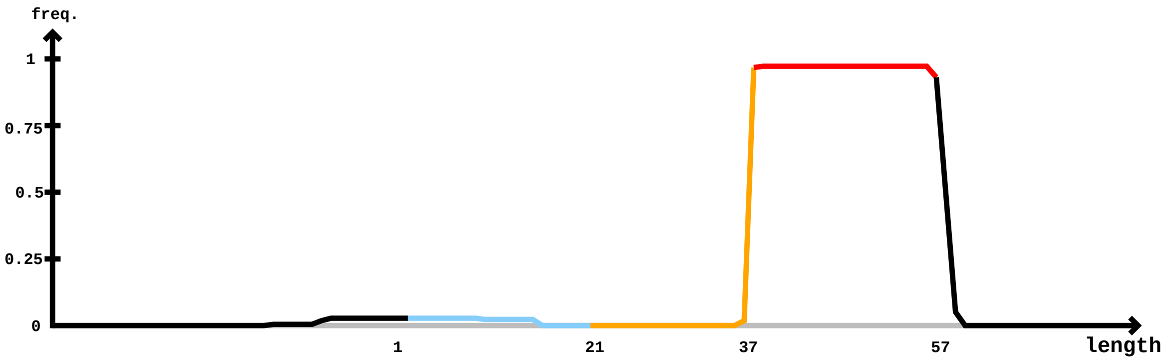


```
                                         Star                                         Mature
5'- ugaagcacaaaaagcuuauguauuggguuggccaaaauguucguucagguuuuuccauacacuccuauggcaaaaucugaaugaacuuuuugcccauuugaacucaaucac   -3'    exp
    ..((((.......)))).((.((((((((((.(((((.(((((((((((((((.(((((......))))).))))))))))))))).))))).))).....)))))))))).   reads      mm        sample
    .....................auuggguuggccaaaauguucg....................................................................   1          0         seq
    ..........................guuggccaaaauguucguucagg..............................................................   3          0         seq
    ...........................uuggccaaaauguucguucagg..............................................................   1          0         seq
    ...........................uuggccaaaauguucUuucagg..............................................................   1          1         seq
    ......................................................................caaaaucugaaugaacuuuuu....................   3          0         seq
    ......................................................................caaaaucugaaugaacuuuuug...................   1          0         seq
    .......................................................................aaaaucugaaugaacuuuu.....................   9          0         seq
    .......................................................................aaaaucugaaCgaacuuuuu....................   8          1         seq
    .......................................................................aaaCucugaaugaacuuuuu....................   3          1         seq
    .......................................................................aaaaucugaauAaacuuuuu....................   1          1         seq
    .......................................................................aaaaucAgaaugaacuuuuu....................   1          1         seq
    .......................................................................aaaaucugaaugaacuuuuA....................   6          1         seq
    .......................................................................aaaaucugaaugaacuuuuu....................   126        0         seq
    .......................................................................aaaaucugaaugaacuuuuug...................   36         0         seq
    .......................................................................aaaaucugaaugaacuuuuuU...................   6          1         seq
    .......................................................................aaaaucugaaugaGcuuuuugc..................   1          1         seq
    .......................................................................aaaaucugaaugaacuuuuugc..................   9          0         seq
    ........................................................................aaaucugaaugaacuuuuugc..................   1          0         seq
```
